# A New Human Genome Sequence Paves the Way for Individualized Genomics

**DOI:** 10.1371/journal.pbio.0050266

**Published:** 2007-09-04

**Authors:** Liza Gross

Just six years ago, two draft versions of the human genome were published, an achievement widely hailed as one of the most audacious scientific undertakings in history. Both of these versions are composite sequences derived from the haploid genomes—the single set of 23 chromosomes packaged into the sperm or egg of each parent—of (mostly) anonymous donors. But now, one of the principals behind the private human genome initiative has taken the next logical, albeit risky, step: sequencing his own genome. J. Craig Venter, whose technical innovations at Celera helped complete the draft sequences far ahead of schedule, published his entire genome in collaboration with 30 colleagues in this issue of PLoS Biology.

By placing his genome in the public domain, Venter runs the risk of divulging intimate personal details, including any current and future genetic markers for disease—a risk that extends to his family. He has done so, in part, to stimulate efforts to develop cheaper sequencing technology and usher in a new era of individualized genomic medicine. Venter has been working with the X Prize Foundation, which has promised US$10 million to the first person who can sequence a genome for US$1,000. Having the complete sequence of an individual human being will also allow scientists to ask different questions about the nature and origin of human genetic variation.

James Watson, the original director of the Human Genome Project and now chancellor of Cold Spring Harbor Laboratory, has also allowed his genome to be sequenced. He received a DVD documenting his personal sequence in a ceremony at Baylor College of Medicine in May 2007. (The report on his genome had not been published at press time.) With these sequences, scientists have a powerful tool for exploring the genetic contribution to human biology and disease risk. For example, the International HapMap Project maintains a catalog of common genetic variants (single-base variations called SNPs) among different populations, which has helped scientists identify gene variants (or alleles) associated with increased risk of diabetes and other complex diseases. (Neighboring SNPs that are inherited together are compiled into haplotypes, hence the name HapMap.)

**Figure:**
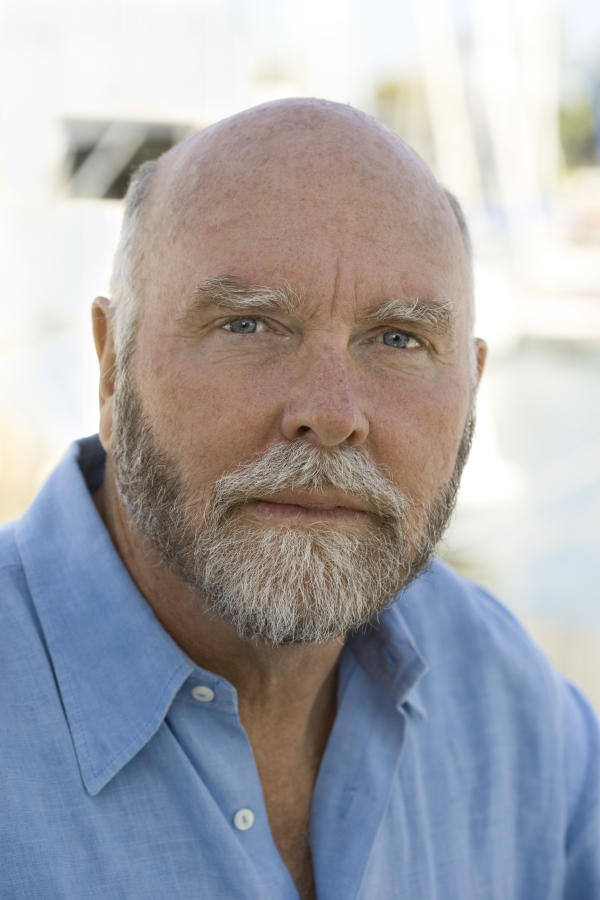
J. Craig Venter (above) led the effort, and donated his DNA, to sequence and assemble the complete genome of a single individual.

But such studies can’t detect rare disease-related alleles or those that reflect individual idiosyncrasies. What’s more, it is the complex interactions between different alleles (contributed by each parent), their regulatory elements, and a person’s environment that determines an individual’s physical characteristics (also known as phenotype) and disease risk. Sequencing both sets of chromosomes from an individual—the diploid genome—who is willing to disclose relevant details about his or her personal life offers the opportunity to correlate genomic variations with a specific phenotype.

Toward this end, Venter donated his blood for DNA extraction and answered questions about his family and medical history, personality, and physical traits. To assemble Venter’s genome (which the researchers called “HuRef”), the researchers modified the random shotgun sequencing approach that Venter used to produce the draft human genome. Briefly, the shotgun sequencing approach randomly shreds genetic material into millions of fragments, called “reads,” each of which is sequenced and then reassembled using a computer (based on sequence similarity), which matches up overlapping reads and merges them into longer sequences. By refining the software algorithms of the computer assembler (to respect the distinct paternal allelic contributions) and increasing the number of times they repeated the sequencing (to enhance data accuracy), the researchers decreased the number of gaps in the assembly to produce a high-quality draft diploid genome sequence. Assembling the sequences in the proper order and location along the chromosomes was guided in part by comparing the HuRef sequence with the composite human sequence assemblies.

To characterize the extent and type of variation in an individual genome, the researchers first identified differences between the maternal and paternal chromosomes (that is, heterozygous alleles) and then compared the HuRef sequence to the latest version of the composite human genome sequence (known as NCBI 36). As one would expect, very large regions of HuRef matched up with the NCBI reference sequence, simplifying the task of spotting variation. This comparison identified over 4 million variants, 68% of which matched those in the central public SNP database (dbSNP). Variants came mostly in the form of SNPs, but another type of genomic alteration—nucleotide insertions and deletions (called indels)—contributed a surprising proportion of observed variation. Although indels are far less common than SNPs, since they can involve several nucleotides, they actually account for nearly three-quarters of the variant nucleotides detected.

Because it’s easier to use haplotypes than SNPs to identify disease risk in association studies—and would likely prove easier in identifying an individual’s risk—the researchers used a novel computational approach to infer haplotypes in the HuRef sequence using the identified DNA variants and the underlying genome assembly structure. Their method generated haplotypes that were not only “strongly consistent” with those found in the HapMap data but also extended the size of inferred haplotype blocks by an order of magnitude, validating the accuracy of the approach.

What does the researchers’ “initial foray into individualized genomics” tell us? Genetic variation between the chromosome sets inherited from one’s parents is far greater than previously recognized, when the relatively obscure “indels” are taken into account. And genetic differences between individuals, the researchers suspect, may be 5-fold higher than prior estimates. The predictive power of individualized genomics, they argue, will depend on gathering far more genomic data from many more individuals. Until then, an individual’s genome sequence will work best in predicting risk for diseases associated with single-gene mutations, like Huntington disease or Fragile X syndrome.

The human genome sequences—the composite sequences and now this individual genome—provide a powerful tool for reconstructing the molecular agents of human evolution, development, physiology, medicine, and individuality. No one expects to understand what makes a person tick by looking at their genome. But as researchers amass more and more individual genomes annotated with personal traits, they’ll be able to test the relative contributions of genes and environment and provide a more informed account of what makes us who we are.

You can explore Venter’s genome yourself, by viewing an interactive poster of his complete genome (10.1371/journal.pbio.0050254.sd001).

